# Supplementary material for: Effects of Anti-Fibrotic Drugs on Transcriptome of Peripheral Blood Mononuclear Cells in Idiopathic Pulmonary Fibrosis
Source: Int J Mol Sci. 2024 Mar 28;25(7):3750. doi: 10.3390/ijms25073750 (PMC11011476; doi:10.3390/ijms25073750)
Supplement: Supplementary file 1 [file ijms-25-03750-s001.zip › captions for supplementary files.pdf]

Figure S1: Details of Figure 1c.
Figure S2: Details of Figure 2c.
Figure S3: Comparison of gene expression levels before and after pirfenidone (PFD) administration using real-time quantitative PCR. (a) *CCNE1*, (b) SERPINE1. We performed qRT-PCR to confirm the potential effects of PFD on the transcriptome signature of PBMCs in patients with IPF. The qRT-PCR of CCNE1 and SERPINE1 showed a trend similar to the transcriptome signature, though not significant because there were only three specimens.
Figure S4: Details of Figure 3c.
Figure S5: Comparison of gene expression levels before and after nintedanib (NTD) administration using real-time qPCR. (a) *ACOT7*, (b) *CCNB2*, (c) *CDK1*, (d) *SERPINE1.* We performed qRT-PCR to confirm the potential effects of NTD on the transcriptome signature of PBMCs in patients with IPF. The qRT-PCR of ACOT7, CCNB2, CDK1, and SERPINE1 showed a trend similar to the transcriptome signature, though not significant because there were only three specimens.

Table S1: Details of oral medication in the patients with IPF.
Table S2: Differential gene expression in PBMCs between idiopathic pulmonary fibrosis (IPF) and. healthy controls (HCs).
Table S3: Enrichment analysis of transcriptome data. Gene Ontology (biological process): IPF vs. HCs.
Table S4: Enrichment analysis of transcriptome data. Gene Ontology (molecular function): IPF vs. HCs.
Table S5: Enrichment analysis of transcriptome data. Gene Ontology (cellular component): IPF vs. HCs.
Table S6: Differential gene expression in PBMCs between before and after PFD administration.
Table S7: Differences in transcriptome levels of molecules related to PFD acting mechanisms.
Table S8: Enrichment analysis of transcriptome data. Gene Ontology (biological process): before vs. after PFD administration.
Table S9: Enrichment analysis of transcriptome data. Gene Ontology (molecular function): before vs. after PFD administration.
Table S10: Enrichment analysis of transcriptome data. Gene Ontology (cellular component): before vs. after PFD administration.
Table S11: Differential gene expression in PBMCs between before and after NTD administration.
Table S12: Differences in transcriptome levels of molecules related to NTD acting mechanisms.
Table S13: Enrichment analysis of transcriptome data. Gene Ontology (biological process): before vs. after NTD administration.
Table S14: Enrichment analysis of transcriptome data. Gene Ontology (molecular function): before vs. after NTD administration.
Table S15: Enrichment analysis of transcriptome data. Gene Ontology (cellular component): before vs. after NTD administration.
Table S16: RPM data of the representative gene expression levels**.**
