# Supplementary figures and images for: Effects of Anti-Fibrotic Drugs on Transcriptome of Peripheral Blood Mononuclear Cells in Idiopathic Pulmonary Fibrosis
Source: Int J Mol Sci. 2024 Mar 28;25(7):3750. doi: 10.3390/ijms25073750 (PMC11011476; doi:10.3390/ijms25073750)

Supplementary Figure S3

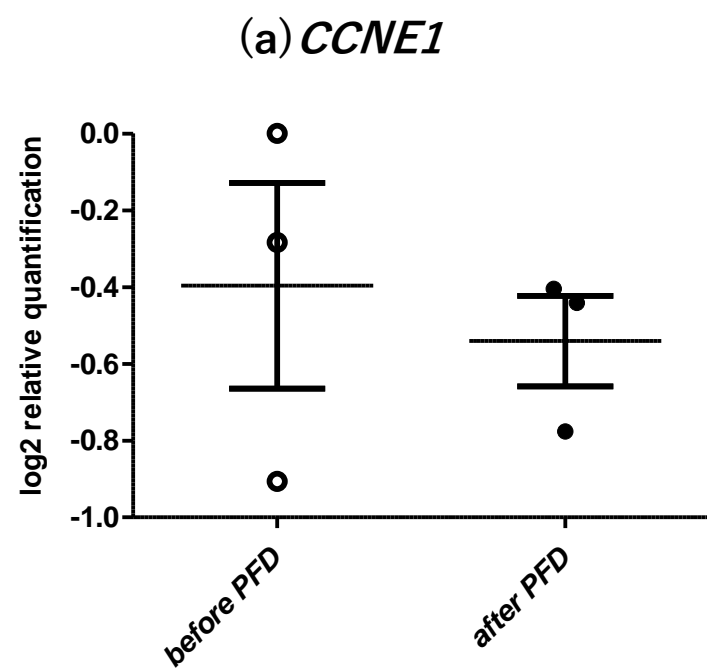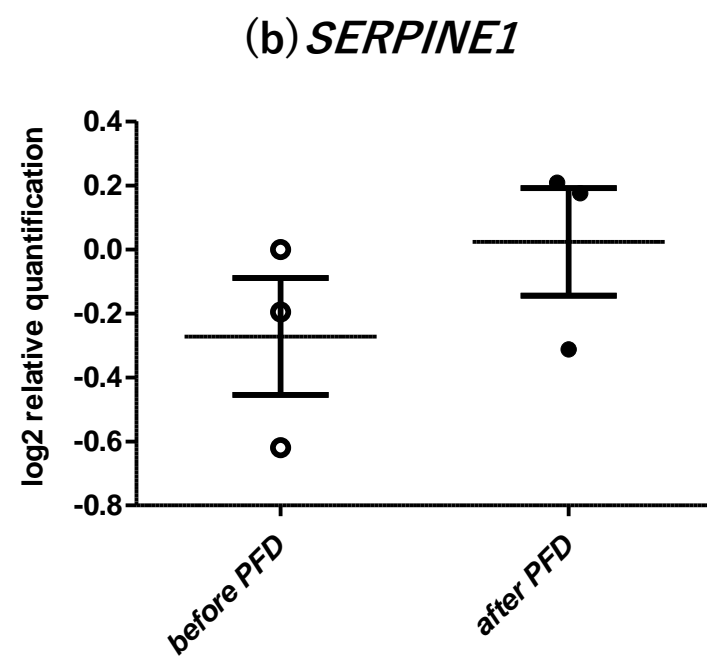

Supplement: Supplementary file 1 [file ijms-25-03750-s001.zip › 022124 Supplementary Figures IPF PBMC/032524 Supplementary Figure S3.pdf]

Supplementary Figure S5

(a) *ACOT7*

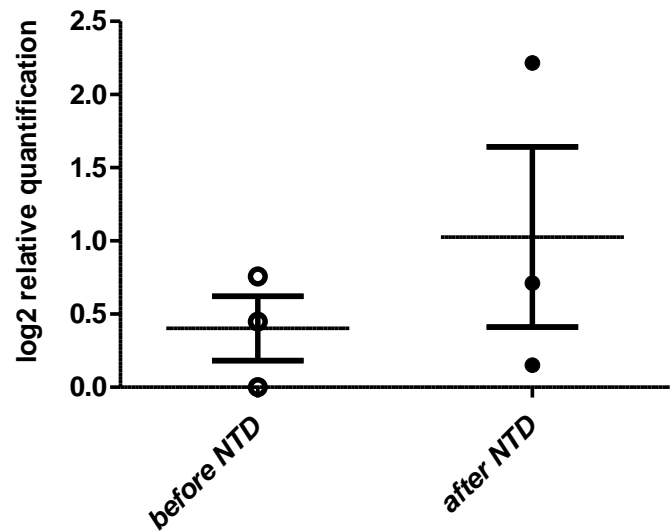

(b) *CCNB2*

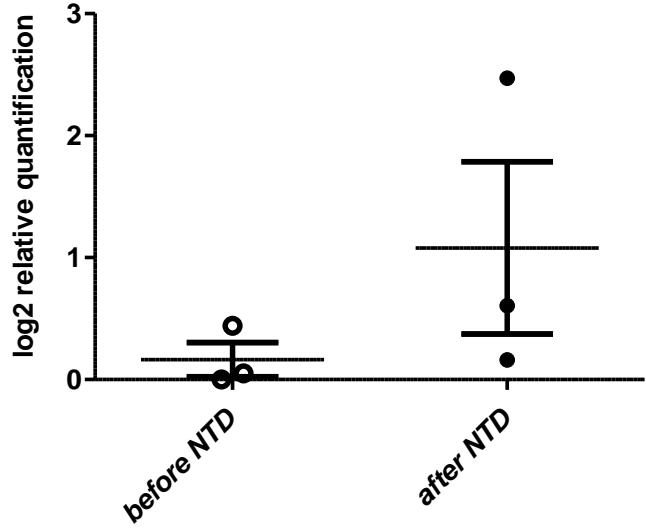

(c) *CDK1*

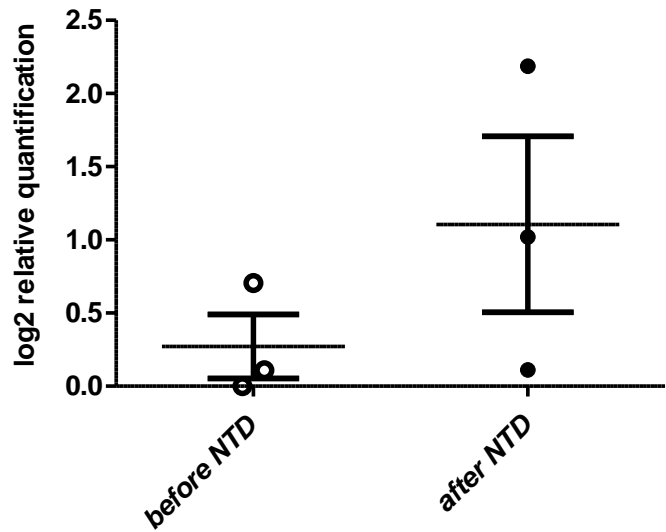

(d) *SERPINE1*

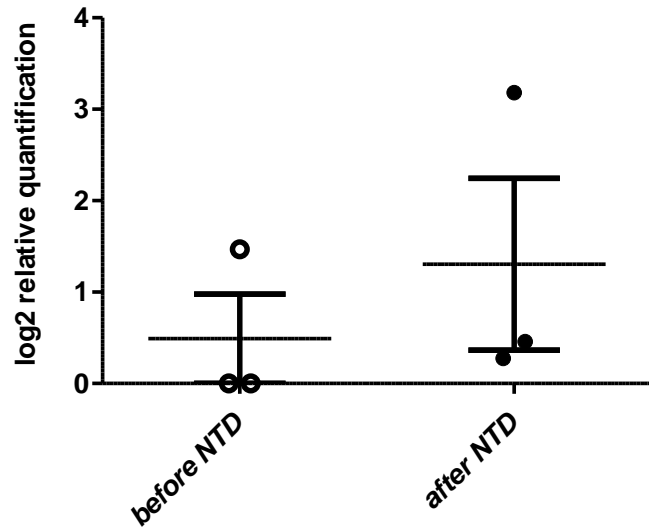

Supplement: Supplementary file 1 [file ijms-25-03750-s001.zip › 022124 Supplementary Figures IPF PBMC/032524 Supplementary Figure S5.pdf]

Supplementary Figure S1

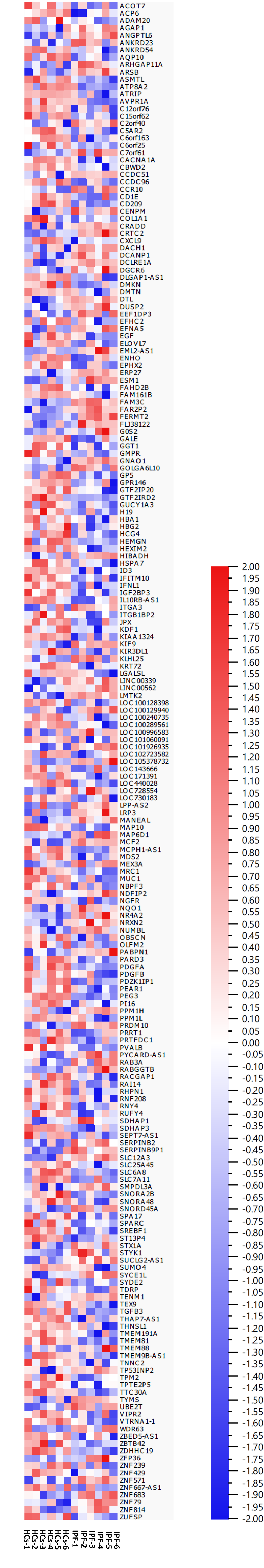

Supplement: Supplementary file 1 [file ijms-25-03750-s001.zip › 022124 Supplementary Figures IPF PBMC/121323 Supplementary Figure S1.pdf]

Supplementary Figure S2

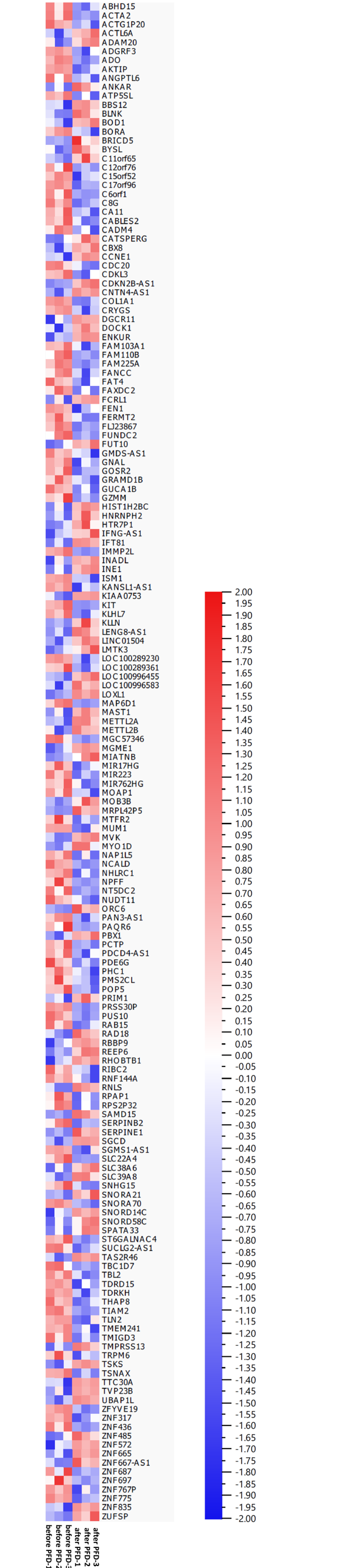

Supplement: Supplementary file 1 [file ijms-25-03750-s001.zip › 022124 Supplementary Figures IPF PBMC/121323 Supplementary Figure S2.pdf]

Supplementary Figure S3

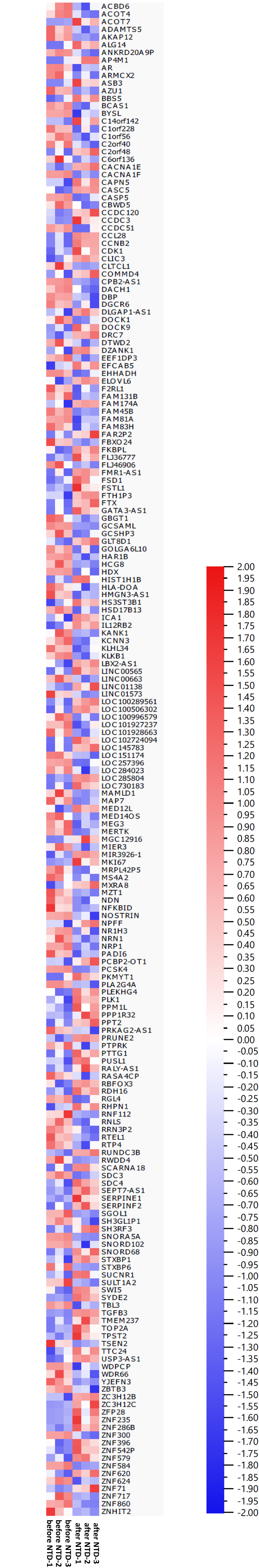

Supplement: Supplementary file 1 [file ijms-25-03750-s001.zip › 022124 Supplementary Figures IPF PBMC/121323 Supplementary Figure S4.pdf]
